# An outbreak caused by the SARS-CoV-2 Delta (B.1.617.2) variant in a care home after partial vaccination with a single dose of the COVID-19 vaccine Vaxzevria, London, England, April 2021

**DOI:** 10.2807/1560-7917.ES.2021.26.27.2100626

**Published:** 2021-07-08

**Authors:** Sarah V Williams, Amoolya Vusirikala, Shamez N Ladhani, Elena Fernandez Ruiz De Olano, Nalini Iyanger, Felicity Aiano, Kelly Stoker, Guduru Gopal Rao, Laurence John, Bharat Patel, Nick Andrews, Gavin Dabrera, Mary Ramsay, Kevin E Brown, Jamie Lopez Bernal, Vanessa Saliba

**Affiliations:** 1 National Infection Service, Public Health England, London, United Kingdom; 2 London Coronavirus Response Centre, Public Health England, London, United Kingdom; 3 London North West University Healthcare NHS Trust, London, United Kingdom

**Keywords:** COVID-19, SARS-CoV-2, Delta variant, vaccine effectiveness, care home outbreaks, Vaxzevria, ChAdOx1 vaccine, B.1.617.2 variant

## Abstract

We investigated a COVID-19 outbreak of the SARS-CoV-2 Delta variant of concern in a London care home, where 8/21 residents and 14/21 staff had received a single dose of Vaxzevria (ChAdOx1-S; AstraZeneca) vaccine. We identified 24 SARS-CoV-2 infections (16 residents, 8 staff) among 40 individuals (19 residents, 21 staff); four (3 residents, 1 staff) were hospitalised, and none died. The attack rate after one vaccine dose was 35.7% (5/14) for staff and 81.3% (13/16) for residents.

In April 2021, a coronavirus disease (COVID-19) outbreak occurred at a care home in London, England, affecting both residents and staff, most of whom had received a single dose of Vaxzevria (ChAdOx1-S; AstraZeneca, Cambridge, UK) vaccine. Whole genome sequencing confirmed the outbreak was caused by the severe acute respiratory syndrome coronavirus 2 (SARS-CoV-2) Delta variant (Phylogenetic Assignment of Named Global Outbreak (Pango) lineage designation B.1.617.2). The outbreak investigation and SARS-CoV-2 serology were used to understand the impact of vaccination against infection and hospitalisation among residents and staff.

## Affected care home

The care home had 21 residents (median age: 81 years; interquartile range (IQR): 78–86; 12 women) and 21 permanent members of staff (median age: 49 years; IQR: 42–59; 16 women). When the outbreak started, the home did not routinely employ agency staff.

Since summer 2020, the care home conducted screening of staff with rapid lateral flow tests twice weekly and nasal-pharyngeal SARS-CoV-2 RT-PCR weekly; screening of residents with SARS-CoV-2 RT-PCR was conducted monthly [1,2]. If staff and residents developed COVID-19 symptoms or were hospitalised for any reason, they were also tested for SARS-CoV-2 by RT-PCR. The care home had not previously experienced a COVID-19 outbreak, defined as two or more SARS-CoV-2-positive cases within a 2-week period [2].

### Ethical statement

PHE has legal permission, provided by Regulation 3 of the Health Service (Control of Patient Information) Regulation 2002, to process patient confidential information for national surveillance of communicable diseases. The Investigation Protocol was reviewed and approved by the PHE Research Ethics and Governance Group (REGG) (Reference NR0252). Verbal consent for testing was obtained by care home managers from staff members and residents or their next of kin as appropriate.

## Outbreak evolution

At the start of the outbreak, the index COVID-19 case was a symptomatic staff member who tested positive with a lateral flow test in early April 2021 and tested positive with RT-PCR 3 days later; the staff member had received the first Vaxzevria vaccine dose in January 2021. The staff member reported a household contact who had recently returned from India 7 days before the staff member tested positive; the household contact had tested positive by RT-PCR 5 days before the staff member’s positive test. An additional four COVID-19 cases at the care home were identified 6 and 7 days after the index case. These residents were admitted to the hospital, three with COVID-19 symptoms and one with an unrelated medical condition. 

**Figure 1:**
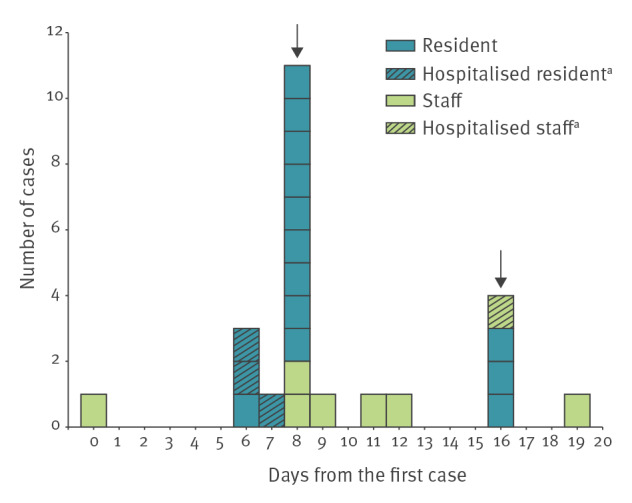
Epidemic curve of a COVID-19 outbreak caused by the SARS-CoV-2 Delta variant in care home residents and permanent members of staff, London, England, April 2021 (n = 24)

COVID-19: coronavirus disease; SARS-CoV-2: severe acute respiratory syndrome coronavirus 2.

An outbreak was declared and, as per national guidance [2], testing of all care home residents and staff was performed twice (on day 8 and 16) after the positive result in the index case. Because of concerns about the number of cases, whole care home testing was repeated again on day 25. In total, the whole home testing identified 15 cases, while four additional cases were detected in staff members outside whole home testing. The last case was in a staff member on day 19, as shown in the epidemic curve (Figure 1). Among 40 swabbed individuals (19 residents, 21 staff), there were 24 cases (16 residents, eight staff); two residents did not consent to PCR testing. Of the cases, four were hospitalised for COVID-19 (three residents, one staff member) for 1–4 days, two required supplemental oxygen, and none required intensive care or died within 28 days of diagnosis (Table 1). A case was defined as a person with SARS-CoV-2 infection confirmed by RT-PCR.

Arrows indicate whole care home testing for SARS-CoV-2.

^a^ Residents and staff hospitalised with COVID-19-related symptoms. Those hospitalised because of other causes are not included.

**Table 1:** Characteristics of cases and non-cases in a COVID-19 outbreak caused by the SARS-CoV-2 Delta variant in a care home, London, England, April 2021 (n = 42)

Characteristics	Overall (n = 42)		COVID-19 cases (n = 24)		Non-cases^a^ (n = 18)	
	n	%	n	%	n	%
Resident	21	50	16	66.7	5	27.8
Staff	21	50	8	33.3	13	72.2
**Sex**						
Men	14	33.3	9	37.5	5	27.8
Women	28	66.7	15	62.5	13	72.2
**Age group (years)**						
30–39	5	11.9	2	8.3	3	16.7
40–49	6	14.3	2	8.3	4	22.2
50–59	6	14.3	4	16.7	2	11.1
60–69	6	14.3	2	8.3	4	22.2
70–79	5	11.9	4	16.7	1	5.6
≥ 80	14	33.3	10	41.7	4	22.2
**Ethnicity **						
White	12	28.6	10	41.7	2	11.1
Black	2	4.8	2	8.3	0	0
Asian	10	23.8	8	33.3	2	11.1
Mixed	0	0	0	0	0	0
Other	3	7.1	3	12.5	0	0
Unknown	15	35.7	1	4.2	14	77.8
**Symptomatic **						
Yes	10	23.8	10	41.7	0	0
**Hospitalised^b^**						
Yes	4	9.5	4	16.7	0	0
**Vaccination status^c^**						
0–20 days post dose 1	0	0	0	0	0	0
≥ 21 post dose 1	2	4.9	2	8.3	0	0
0–13 post dose 2	28	68.3	16	66.7	12	70.6
≥ 14 post dose 2	3	7.3	3	12.5	0	0
Unvaccinated	8	19.5	3	12.5	5	29.4

COVID-19: coronavirus disease; SARS-CoV-2: severe acute respiratory syndrome coronavirus 2.

^a^ Non-cases are defined as residents or permanent staff with negative SARS-CoV-2 RT-PCR tests during the outbreak period (n = 16) or refused a test (n = 2).

^b^ Residents and staff hospitalised with COVID-19-related symptoms. Those hospitalised because of other causes are not included.

^c^ Vaccination with one or two doses of Vaxzevria (ChAdOx1-S; AstraZeneca, Cambridge, United Kingdom) COVID-19 vaccine (n= 41). One staff member received dose 1 of the Comirnaty COVID-19 (BNT162b2 mRNA, BioNTech-Pfizer, Mainz, Germany/New York, United States) vaccine more than 21 days ago, but not included with those in the ‘≥ 21 post dose 1’ vaccination status category.

The outbreak was declared over in mid-May 2021, 28 days after the last case was detected. No further cases were detected following whole care home PCR testing.

## Vaccination status

All residents and all staff members who were vaccinated, except one, had received the Vaxzevria vaccine. The first dose was given on 14 January 2021 (18/21 residents, 14/21 staff) and the second dose was given on 1 April 2021 (18/21 residents, 13/21 staff), the same day that the index case was diagnosed with COVID-19. Exceptions include one resident who received a first dose of Vaxzevria vaccine on 12 March 2021 and one staff member who received a first dose of Comrinaty (BNT162b2 mRNA, BioNTech-Pfizer, Mainz, Germany/New York, United States (US)) on 16 February 2021.

## Genomic analysis

Whole genome sequencing was performed for RT-PCR-positive swabs that were tested at Public Health England (PHE) Colindale Virus Reference Department and had a cycle threshold (Ct) value of < 35. Sequencing was successful for 16/24 samples: eight were confirmed and eight were probable as the Delta variant.

## Attack rates in Vaxzevria-vaccinated staff and residents

The attack rate after the first vaccine dose was 35.7% (5/14) for staff and 81.3% (13/16) for residents (Table 2). Because of the small size of the care home, vaccine effectiveness (VE) was not calculated. Serological testing was offered to all care home staff and residents 28 days after the last case (using the Roche Elecsys Anti-SARS-CoV-2 serology assay for the detection of anti-SARS-CoV-2 N antibodies and the Roche Elecsys Anti-SARS-CoV-2 S serology assay for the detection of anti-SARS-CoV-2 S antibodies, Roche Diagnostics Limited, West Sussex, United Kingdom [3,4]). Of the 24 SARS-CoV-2-positive cases, all 11 who were serologically tested had received at least one vaccine dose; all were both N antibody-positive (consistent with previous infection) and S antibody-positive (consistent with previous infection and/or vaccination). S antibody titres were high (> 6,000 AU/ml) and consistent with a combination of vaccination and natural COVID infection.

**Table 2:** Attack rates in Vaxzevria-vaccinated and -unvaccinated residents and staff during a COVID-19 outbreak caused by the SARS-CoV-2 Delta variant in a care home, London, England, April 2021 (n = 36)

Cases	Attack rates				Reduction rate^c^
	Vaccinated^a^		Unvaccinated^b^		
	n/N	%	n/N	%	
Staff	5/14	35.7	2/5	40	10.7
Residents	13/16	81.3	1/1	100	18.7

COVID-19: coronavirus disease; SARS-CoV-2: severe acute respiratory syndrome coronavirus 2.

^a^ Only those who have received 1 dose or < 14 days after dose 2 of Vaxzevria (ChAdOx1-S; AstraZeneca, Cambridge, United Kingdom) vaccine.

^b^ Excludes non-cases who had antibodies from previous SARS-CoV-2 infection based on serology.

^c^ Reduction rate = (AR unvaccinated - AR vaccinated)/AR unvaccinated x 100 where AR is attack rate.

Residents who did not consent to PCR testing were excluded.

Serology was performed on 10 of the 18 non-cases; all had received at least one Vaxzevria vaccine dose. Of these, five of the non-cases were N-negative and only had S anti-SARS-CoV-2 antibodies, consistent with previous vaccination and no evidence of previous infection. The other five non-cases had both N and S antibodies detected, three of whom had antibody titres suggestive of previous COVID-19 infection only, whereas 2 had titres suggestive of natural infection and vaccination.

## Discussion and conclusion

More than a year into the coronavirus disease (COVID-19) pandemic, new variants continue to emerge and spread rapidly across the continents. In England, the SARS-CoV-2 Delta variant, initially identified in India, was first detected in London and the North West of England in late March 2021 and declared a variant of concern on 6 May 2021 [5]. 

Care homes have been disproportionately affected by the pandemic, with high fatality rates reported among elderly people [6,7]. Care home residents and staff were, therefore, prioritised for vaccination as soon as the first COVID-19 vaccines became available [8]. Clinical trial data at the time indicated that a single dose of the Comirnaty (BNT162b2 mRNA, BioNTech-Pfizer, Mainz, Germany/New York, United States (US)) vaccine was estimated to provide 89% protection from symptomatic disease [9] compared with 95% for two doses given 3 weeks apart [10]. Based on these early clinical trial data indicating rapid protection after a single dose of the Comirnaty vaccine, the United Kingdom (UK) opted for an extended interval of up to 12 weeks between the two-dose schedule for COVID-19 vaccines to accelerate the rollout of the first dose of the vaccine to those at highest risk [8]. Moreover, clinical trials with the Vaxzevria vaccine demonstrated a better boost with longer intervals between vaccine doses [11]. Real-world data have demonstrated high effectiveness – especially in preventing hospitalisations and deaths – with a single dose of either vaccine, even with the more transmissible Alpha (B.1.1.7) variant [12,13] or in high risk settings such as care homes [14]. 

The Delta variant has emerged more recently and so there are limited data on the COVID-19 VE against this variant, especially in high risk populations. A recent preprint reported VE against symptomatic disease to be lower after one dose of either Comirnaty or Vaxzevria vaccine for Delta variant cases (33.5%; 95% confidence interval (CI): 20.6–44.3) compared with Alpha variant cases (51.1%; 95% CI: 47.3–54.7) [15]. However, after two doses, VE increased to 87.9% (95% CI: 78.2–93.2) for Comirnaty and 59.8% (95% CI: 28.9 to 77.3) for Vaxzevria [15].

The high attack rates in this care home outbreak, especially among partially vaccinated residents, is consistent with lower protection against SARS-CoV-2 infection in residents and staff who had received only one dose of the Vaxzevria vaccine within 3 months. Reassuringly, though, hospitalisation was uncommon and there were no deaths, providing some evidence that a single dose of vaccine may be protective against severe disease following infection with the Delta variant. Vaccination with the second dose was coincidentally arranged at the care home on the same day the index case tested positive and it is possible that this helped control the outbreak more quickly. Our findings are supported by recent research that reported VE against hospitalisation of a single dose of Vaxzevria vaccine for the Delta variant as 71% (95% CI: 51–83) compared with 76% (95% CI: 61–85) for the Alpha variant with effectiveness increasing to 92% (95% CI: 75–97) for the Delta variant after two doses of the vaccine [16].

There may be concern that the attack rates are based on very small numbers and so these results should not be over-interpreted as they involve a single outbreak in a small care home. It was not possible to obtain baseline serology samples on staff and residents at the start of the outbreak. In addition, serology samples at 28 days after the conclusion of the outbreak were only available on a subset of cases and non-cases. As routine testing in care homes was not available until the summer of 2020 [1], it is possible that previously asymptomatic infections during the first wave of the pandemic may have conferred some protection against reinfection for some non-cases who were reported as unvaccinated. This would lead to an under-estimation of the apparent difference the vaccine made on attack rates.

By this point, residents and permanent staff in care homes in the UK should all have been offered two vaccine doses. However, given that Delta is the dominant UK variant as of June 2021 [17], it is critical that high uptake of both doses is achieved, especially among staff, since uptake remains suboptimal in some regions [18]. Countries that have opted to extend the interval between two vaccine doses should consider offering the second dose earlier to care home residents and staff, in the context of the circulating Delta variant.
